# Short-term effect of temperature and precipitation on the incidence of West Nile Neuroinvasive Disease in Europe: a multi-country case-crossover analysis

**DOI:** 10.1016/j.lanepe.2024.101149

**Published:** 2024-12-04

**Authors:** Giovenale Moirano, Chloe Fletcher, Jan C. Semenza, Rachel Lowe

**Affiliations:** a Barcelona Supercomputing Center (BSC), Barcelona, Spain; b Department of Medical Sciences, University of Turin, Turin, Italy; c Department of Medicine & Life Sciences, Universitat Pompeu Fabra, Barcelona, Spain; d Heidelberg Institute of Global Health, University of Heidelberg, Heidelberg, Germany; e Department of Public Health and Clinical Medicine, Section of Sustainable Health, Umeå University, Umeå, Sweden; f Catalan Institution for Research and Advanced Studies (ICREA), Barcelona, Spain; g Centre on Climate Change & Planetary Health and Centre for Mathematical Modelling of Infectious Diseases, London School of Hygiene & Tropical Medicine, London, UK

**Keywords:** West Nile Virus, West Nile Neuroinvasive Disease, Meteorological factors, Climate-sensitive disease, Case-crossover study design, Short-term effects

## Abstract

**Background:**

In recent years, Europe has experienced several outbreaks of West Nile Virus (WNV), a mosquito-borne pathogen. This study aims to quantify the impact of weekly mean temperature and cumulative precipitation on human cases of West Nile Neuroinvasive Disease (WNND), to assess the feasibility of climate-informed early warning systems for severe forms of WNV infection.

**Methods:**

Using a space-time-stratified case-crossover design, the short-term effects of meteorological factors on WNND cases reported in Europe from 2014 to 2022 were examined. Distributed lag nonlinear models were implemented in conditional logistic regressions to assess the delayed and nonlinear effects of temperature and precipitation on WNND risk as well as to estimate the Attributable Fraction (AF) of cases to extreme values of the two meteorological factors.

**Findings:**

Between 2014 and 2022, Europe reported 3437 WNND cases. Both meteorological factors recorded in the 8 weeks before symptom onset showed positive and delayed effects on WNND risk. The strongest effect was found for weekly mean temperatures at 2 weeks lag (Odds Ratio (OR): 1.15; 95% Confidence Interval (CI) 1.12–1.19) and for weekly cumulative precipitation at 3 weeks lag (OR: 1.12; 95% CI 1.09–1.16). Of all WNND cases analyzed, 36.4% (95% CI, 31.3%–40.3%) could be attributed to weekly mean temperatures exceeding the 25 °C, while 13.1% (95% CI, 9.5%–16.4%) to weekly cumulative precipitations exceeding 40 mm.

**Interpretation:**

These findings emphasize the significance of short-term variations in temperature and precipitation in driving WNND incidence in Europe. Meteorological factors can be used to operationalize early warning systems to reduce the disease burden from WNV infections, which are continually increasing across the continent.

**Funding:**

10.13039/100018693European Union’s Horizon Europe research and innovation programme.

**Research in context**

**Evidence before this study**

We searched Scopus and PubMed using the terms “temperature”, “precipitation”, “West Nile Virus”, “WNV”, “West Nile Neuroinvasive Disease”, “WNND” for articles in English from database inception until Feb 29, 2024. We identified a systematic review published in 2023 focusing on environmental, climatic, and meteorological factors related to WNV circulation in European and Mediterranean countries. Most of the studies identified in the review addressed the effect of temperature and precipitation on vector abundance and WNV prevalence in vectors or reservoir hosts. Among the 19 studies assessing the role of temperature on human disease, 12 demonstrated a positive effect of temperature on the disease risk. However, most studies evaluated the role of climatic factors, such as seasonal anomalies, in driving interannual variability in disease incidence. Few studies focused on the short-term effects of temperature in driving the within-year incidence of the disease (4 studies) and mainly focused on one or few European countries. Among the 8 studies focusing the role of precipitation on human disease, 5 studies did not find any evidence of an effect. Most of these studies were focused on a single country and not targeting the short-term effects of precipitation on WNV incident cases.

**Added value of this study**

To our knowledge, this is the first study to assess short-term effects of variations in meteorological conditions on WNND incidence (e.g., within the 2 months preceding reported WNND cases) across all Europe over a 9-year period (2014–2022). We performed statistical analyses to estimate the lag-effects, the exposure-response functions, and the health impacts of both weekly average temperature and cumulative precipitation. We found that temperature has a positive effect on WNND risk peaking after 2 weeks, while precipitation has a positive and slightly delayed effect peaking after 3 weeks. About 36% of all WNND cases diagnosed in Europe can be attributed to weekly average temperatures exceeding the 25 °C (the 90th percentile for May to October period), while 13% can be attributed to weekly cumulative precipitations exceeding 40 mm (the 90th percentile May to October period).

**Implications of all the available evidence**

This study provides new evidence on the impact of meteorological factors on WNV incident cases strengthening the evidence of the sensitivity of WNV disease to climate variation and suggesting that climate change can increase the risk of WNV transmission in Europe. Quantifying the effects and time lags between meteorological variations and WNV transmission dynamics can help tailor the climate information needed for early warning systems to detect and respond to human disease outbreaks at the beginning of the transmission season. Future research, following a One Health approach should continue to explore the complex interactions between meteorological factors, vector biology, and human behaviours to inform targeted interventions and reduce the burden of WNV infections.

## Introduction

West Nile Virus (WNV) is an RNA virus that is transmitted between wild birds and mosquitoes. Mosquito species from the genus *Culex* (family *Culicidae*) are the main vectors of WNV transmission, while various wild bird species act as reservoir hosts.^1^ Humans are infected with WNV through the bite of an infected mosquito. In humans, the incubation period spans on average from 2 to 6 days, although longer durations have been documented.^2^ Symptoms can vary, with approximately 80% of infected individuals experiencing mild to no symptoms, whereas 20% develop a febrile syndrome known as West Nile Fever (WNF). Less than 1% of infected subjects develop a neurological syndrome, known as West Nile Neuroinvasive Disease (WNND), which is fatal in 1 in 10 cases.^1^

The first documented large-scale epidemic of WNV in Europe was recorded in Romania in 1996.^3^ Since then, geographical expansion has been observed, mainly in southern and central European countries. Over recent years, the number of human cases has increased significantly in Europe, with an exceptional surge of cases recorded in 2018.^4^ The epidemiology of WNV is determined by both biotic and abiotic factors. Biotic factors include the abundance and diversity of reservoir hosts, such as local and migratory birds, and mosquito vectors. Abiotic factors refer to the physical features of the environment such as weather conditions and land use. Meteorological factors influence the ability of mosquitoes to acquire, maintain, and transmit WNV.^5^^,^^6^ The seasonal timing of these conditions is also crucial for a successful transmission cycle in the context of favorable ecological habitats for vectors and hosts. Accordingly, different meteorological, climatic, ecologic, socioeconomic drivers as well as mosquito density and wild bird immunity, have been linked to European WNV outbreaks in the past years.^7^

Concerning the meteorological drivers, entomological studies have suggested that both temperature and precipitation can impact the risk of WNV transmission by affecting both mosquito population density and virus prevalence among competent vectors.^7^ Epidemiological studies have also suggested that climatic drivers such as positive seasonal anomalies (e.g., 3-month averages) in temperature can affect the inter-annual occurrence of human WNV cases among different European countries.^4^^,^^8^^,^^9^ However, the short-term effects of meteorological factors in driving the timing and intensity of WNV outbreaks in Europe have not been systematically evaluated at a European-wide scale. Here we present a high-resolution analysis to assess the short-term impacts of mean weekly temperature and cumulative weekly precipitation on WNND cases reported across Europe between 2014 and 2022, applying a space-time-stratified case-crossover study design.

## Methods

### Data

Data concerning all WNV human cases recorded from 2013 to 2022 in the European Surveillance System, TESSy were provided by the 27 EU member states and six EU-neighboring countries (Albania, Kosovo, Montenegro, North Macedonia, Serbia and Turkey) and released by the ECDC (European Center for Disease Prevention and Control). Anonymized data included the date of symptom onset and diagnosis, the clinical form (asymptomatic infection, WNF, WNND), and the area of residence at the NUTS3 level (mean of 318,000 inhabitants and of 5540 km^2^ of extension per unit). Data were restricted to WNND cases. Given the high proportion of missing information about the clinical form of cases in 2013 (60.28% of reported cases), the study was restricted to WNND cases diagnosed from 2014, where only 0.01% of reported cases were incomplete. Meteorological data were obtained from the ERA5-Land climate reanalysis database produced by the European Centre for Medium-Range Weather Forecasts (ECMWF). We retrieved the gridded daily mean temperature and daily cumulative precipitation for the European continent over the entire study period (2014–2022). Since ERA5-Land provides atmospheric and land-surface variables with spatial resolution of about 9 km × 9 km, daily data were spatially averaged at the NUTS3 level to align with epidemiological data.

### Study design

To estimate the association between lagged meteorological variables and WNND cases across Europe from 2014 to 2022, we conducted an individual space-time-stratified case-crossover study. Case-crossover design is a special case–control design where every case serves as its own control and was originally developed to evaluate the short-term effect of transient exposures on the risk of acute events.^10^ For each case, exposures occurring during the period prior to the event (“hazard period”) are compared to exposures at comparable control periods (“reference periods”). Space-time-stratified sampling schemes match case and control periods within the same geographical areas and relatively short time strata (i.e., calendar month). Since the inference is based on a comparison of exposure distribution within the matching stratum, this study design enables the joint analysis of cases from different areas and time periods while intrinsically controlling for confounding by geographical area, long-time trends (e.g., variability from year to year) and seasonality (variability from month to month). These characteristics make the space-time-stratified case-crossover design particularly suitable for analyzing multilocation data of a rare health outcome.^11^

In our study, we identified control days within the region, month and year of the case day (date of symptom onset), additionally matching on day of the week. Finally, we attributed the weekly mean temperature and cumulative precipitation recorded in the previous 8 weeks to each case and control period, starting from the day prior to the case and control day. Thus, in our study each case served as its own control. Specifically, for each case, we selected control days within the same calendar month and year in the same NUTS3 region, additionally matching for the day of the week. For example, if a case was observed on Friday, May 17th, 2019, in region “*s”*, the control days were other Fridays of May 2019 (i.e., May 3rd, 10th, 24th, and 31st). We then compared exposures occurring within region *“s”* during the 8 weeks preceding each case and control day to infer associations between exposure levels and the outcome.

### Statistical analysis

Descriptive analyses were conducted for both exposures and the outcome under study. We computed the median and the interquartile range for weekly mean temperatures and weekly cumulative precipitations across the main European transmission season, from the first week of May to the last week of October, across all NUTS3 areas that reported WNND cases during the study period. We computed the weekly time series of WNND cases over 9 years across the entire study area and the WNND average incidence rates by NUTS3 area to evaluate the temporal and spatial distribution of the disease in Europe.

The associations between WNND cases with lagged weekly mean temperature and cumulative precipitation were modelled by implementing distributed lag nonlinear models into conditional logistic regression.^12^ We fit a conditional logistic regression model that included delayed and nonlinear exposure response functions for both mean temperature and cumulative precipitation.

The final model was specified as follows:

$$logit\;\left(P\;\left(Y=1|X_{1},X_{2},S\right)\right)=\alpha_{0s}\;+\;f\left(X_{1t},l\right)\;+\;f\left(X_{2t},l\right)$$

where $P(Y=1\;|\;X_{1},X_{2},S)$ is the conditional probability of being a case (Y=1) given the matching stratum S and the exposure history for temperature, $X_{1}$, and precipitation, $X_{2}$. The stratum specific intercept is noted as $\alpha_{0s}$, while $f\left(X_{1t},l\right)$ and $f\left(X_{2t},l\right)$ represent the nonlinear exposure–lag functions for temperature and precipitation, respectively. Different function combinations to model the nonlinear and delayed effects were tested (see Supplementary Materials). The selection of the final functions for both the exposure–response relationship and the lag-effect was based on the Akaike Information Criterion (AIC). In the final model, the temperature effect was modelled with a natural cubic spline with two internal knots at the 33rd and 66th percentiles, while precipitation was modelled with a linear term. For both variables the lag-effect up to 8 weeks was modelled with a basis cubic spline with two internal knots at equally spaced intervals. All estimates of lagged effects for both temperature and precipitation were expressed as Odds-Ratios (OR) and relative 95% Confidence Intervals (CIs).

Using the estimated associations between lagged temperature, precipitation and WNND risk, we calculated the cumulative exposure-response function, i.e., the sum of each specific lag contribution over the whole lag period, which can be interpreted as the overall risk across 8 weeks. To calculate the attributable fraction (AF) of WNND cases to the exposure to temperature and precipitation over the 8-week lag periods we used a backward approach.^13^ For both temperature and precipitation, the ${AF}_{x,t}$ for observation at time *t* was calculated for exposure level *x* using the following formula:

$${AF}_{x,t}=1\;-\;exp\;\left(\overset{L}{\underset{l=l_{0}}{-\sum }}\beta_{x_{t-l},l}\right)$$

${AF}_{x,t}$ indicate the related fractions at time t that can be attributed to previous level of exposure *x* in the period *t – l*_*0*_*, …, t − L*, with *l*_*0*_ and *L* corresponding to the lowest and highest time lags, while the $\beta_{x_{t-l},l}$ refers to the coefficients associated with the level of exposure *x* experienced in the past over the period *t – l*_*0*_*, …, t − L*. The definition provided above can be expressed with regards to different thresholds of exposure *x* when compared to a counterfactual condition of a constant exposure for the whole lag period.^14^ Applying this approach, we computed the 75th, 90th, 95th and 99th percentiles of the distributions observed across all NUTS3 areas with reported WNND cases during the main WNV transmission season in Europe (May–October period) from 2014 to 2022. The counterfactual condition was defined as a constant exposure to the 75th percentile while exposure thresholds were defined using the 90th, 95th and 99th percentiles. Empirical confidence intervals (eCIs) were calculated through Monte Carlo simulations, assuming a multivariate normal distribution of the point estimate and (co)variance matrix obtained from the regression models. All analyses were performed using the “dlnm” and “survival” packages of the R software (version 4.1.2). The AF was calculated by “attrdl” function provided by Gasparrini et al.^13^

In addition, we investigated geographical heterogeneity of the effects including data from 5 countries (Italy, Greece, Romania, Serbia, and Hungary), where the number of cases and the statistical power was sufficient to estimate the country-specific exposure-lag-response function. For the evaluation of heterogeneity, we followed a two-stage approach.^15^ In the first stage, we fitted the conditional logistic model separately for each of the 5 countries and extracted the coefficients and uncertainty measures associated with the effects of both meteorological factors over the 8 weeks. We extracted the coefficients relevant to 1 °C or 10 mm increase above the country-specific 75th percentile of temperature and precipitation recorded during the WNV transmission season (May–October), respectively. In the second stage, coefficients representing estimated associations between the two meteorological factors and WNND incidence for the 5 countries were pooled using a multivariate random effect meta-analytic model.^15^ From the meta-analytic model, we obtained: i) the pooled estimates representing the average relationships of the two meteorological factors among the 5 countries under study, ii) the country-specific best linear unbiased predictions (BLUPs), which represent a weighted average between the country-specific relationship provided by the first-stage regression and the pooled relationship, iii) the I-squared statistic as a measure of heterogeneity of effects across countries.^15^ Analyses were performed using the “dlnm”, “survival”, and “mixmeta” packages of the R software (version 4.1.2).

Finally, as the first sensitivity analysis, we included a cubic basis spline function in the model for the day of the year with seven equally spaced internal knots. This adjustment aimed to account for potential residual seasonal confounding that might persist even if monthly strata are deployed in the time-stratified case-crossover design.^16^ In the second sensitivity analysis, we excluded from the analyses all WNND cases diagnosed in 2018 to explore whether our results were impacted by this epidemiological outlier.^4^ In the third sensitivity analysis, we excluded from the analyses all WNND cases diagnosed in 2022 to explore if our results were impacted by this climatic outlier.^17^

### Role of the funding source

The funders had no role in the study's design, data collection, analysis, interpretation, writing, or the decision to submit the paper for publication.

## Results

**Fig. 1:**
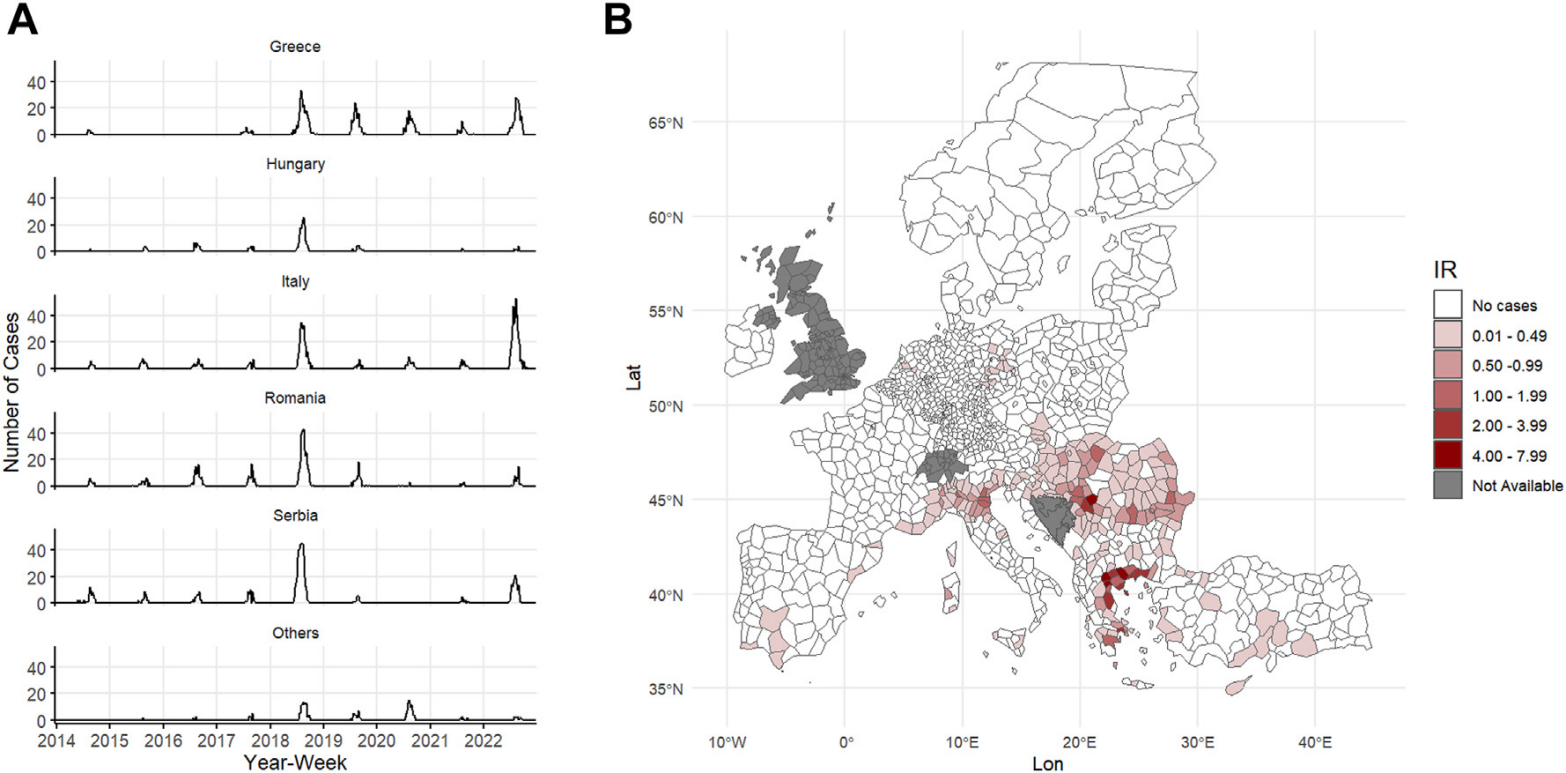
**Spatio-temporal distribution of WNND cases in the study area, 2014–2022**. Panel A: Time series of WNND cases reported in the study area over the 9 years, stratified by country. Y-axis shows the absolute number of WNND cases by week of symptom onset, x-axis shows the time (weeks). Panel B: Spatial distribution of WNND average Incidence Rates per 100,000 person-years at European NUTS3 level; IR: Incidence Rates, Grey areas: data not available.

**Fig. 2:**
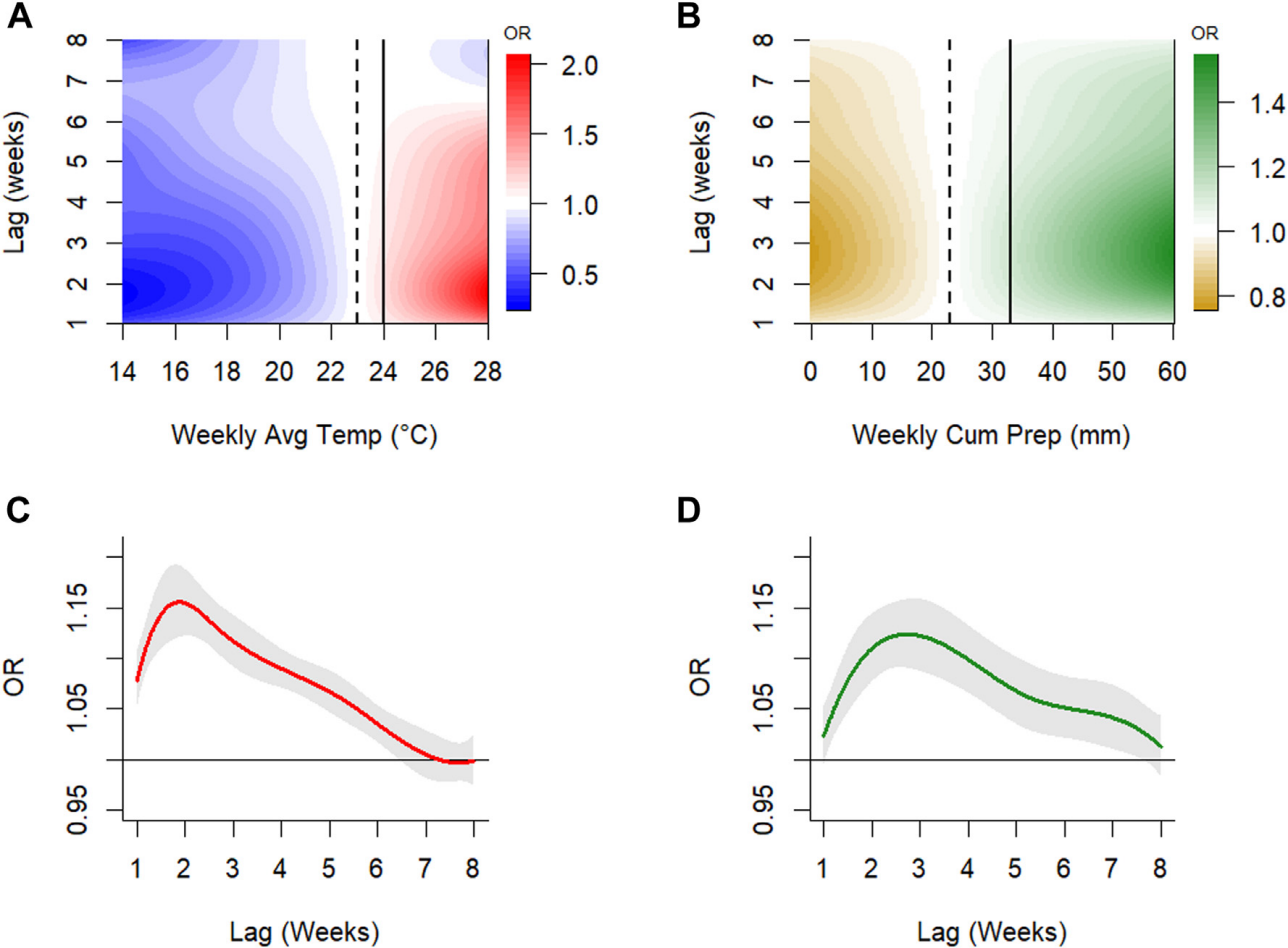
**Association between meteorological factors and WNND cases (N = 3437), 2014–2022**. Panel A: Exposure-lag-response estimates for the weekly mean temperature (°C) and risk of WNND, Dashed line: 75th percentile (23 °C) for weekly mean temperature of the transmission season (May–October period); Continuous Line: 1 °C increase above the 75th percentile. Panel B: Exposure-lag-response estimates for the weekly cumulative precipitation (mm) and risk of WNND, Dashed line: 75th percentile (23 mm) for the weekly cumulative precipitation of the transmission season (May–October period); Continuous Line: 10 mm increase above the 75th percentile. Panel C: Lag-specific Odd-Ratios (OR) and 95% confidence intervals (CIs) for a 1 °C increase (continuous line of Top-left panel) above the 75th percentile (dashed line of Top-left panel) over 8 weeks of lag. Panel D: Lag-specific OR and 95% CIs for a 10 mm increase (continuous line of Top-left panel) above the 75th percentile (dashed line of Top-left panel) over 8 weeks of lag.

Between 2014 and 2022, a total of 3437 WNND cases were reported in Europe. The peak usually occurred during the second week of August, while 95% of the cases occurred between the last week of June and the end of September (Fig. 1). In total, there were 3161 cases diagnosed in Italy, Greece, Serbia, Romania and Hungary, representing 91.9% of all WNND cases observed in the study area (Fig. 1, Table S1). Supplementary Materials (Table S2) present the distributions of the two meteorological parameters for the WNV transmission season (May–October). A positive and delayed effect of weekly mean temperature and cumulative precipitation on WNND risk was observed (Fig. 2, Table S4). The effect on WNND incidence was observed for temperatures recorded 1–6 weeks prior to symptom onset, with the strongest effect observed at lag 2 weeks (OR for 1 °C above the 75th percentile, 23 °C at lag 2: 1.15; 95% CIs: 1.12–1.19). We also observed a positive effect of weekly cumulative precipitation recorded in the weeks prior to symptoms onset on WNND risk, with a more delayed peak effect if compared to temperature (lags 2–7 weeks). The strongest effect was observed at lag 3 weeks (OR for 10 mm above the 75th percentile at lag 3: 1.12; 95% CIs: 1.09–1.16). The exposure-response function at each lag for both meteorological factors are shown in the Supplementary materials (Figures S1 and S2).

**Fig. 3:**
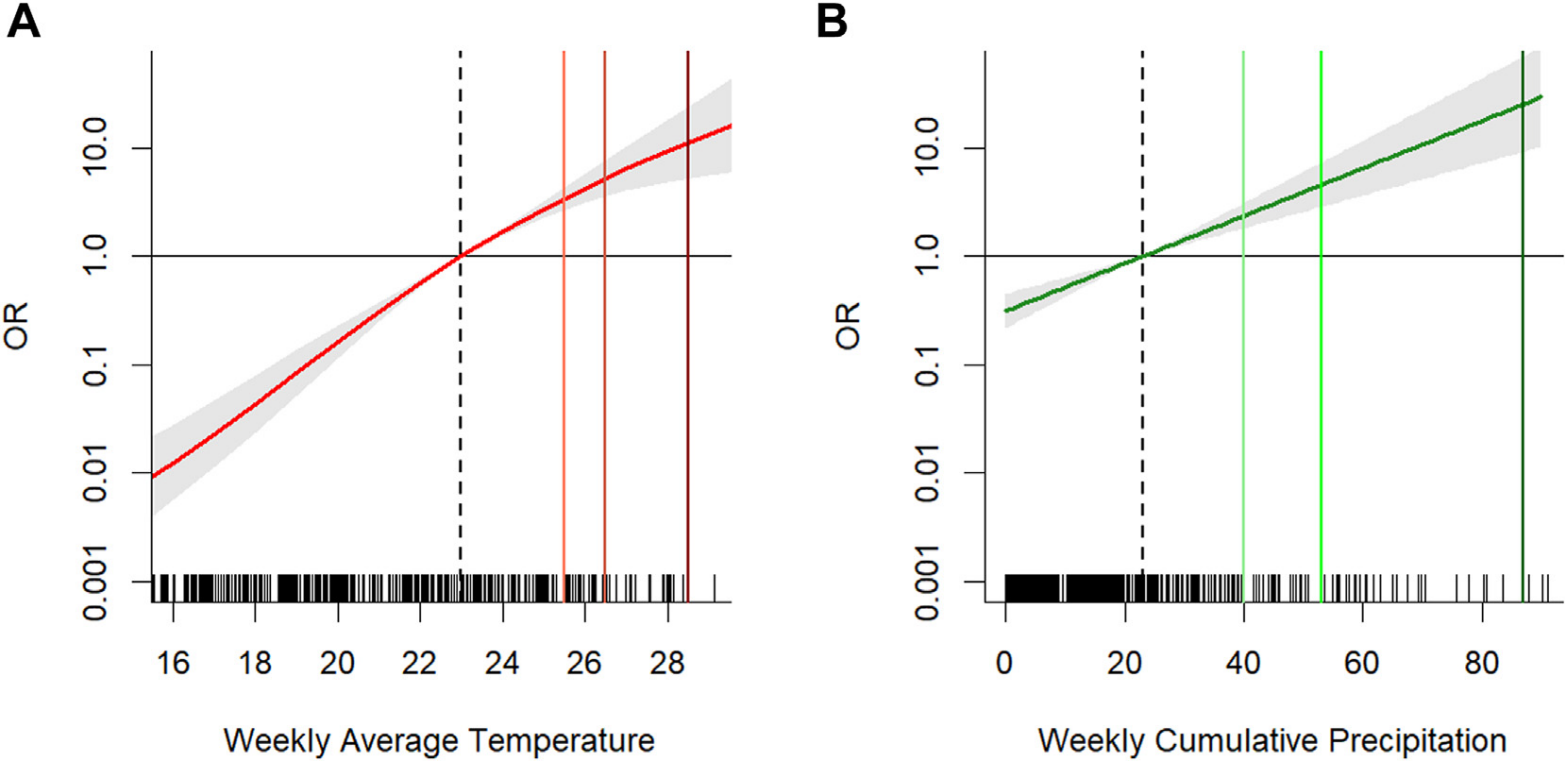
**Cumulative exposure-response functions for the meteorological factors and WNND cases**. Panel A: Cumulative exposure-response function across the 8 weeks for weekly mean temperature (°C); Vertical lines: 75th, 90th, 95th and 99th percentile for the transmission period (May–October period). Panel B: Cumulative exposure-response function across the 8 weeks and weekly cumulative precipitation (mm); Vertical lines: 75th, 90th, 95th and 99th percentile for the transmission period (May–October period).

Fig. 3 represents estimates of the cumulative exposure-response function, representing the sum of each specific lag contribution over the whole lag period (i.e., the overall risk across the 8 weeks). The cumulative OR for a 1 °C increase above the 75th percentile on WNND risk in the following 8 weeks was 1.68 (95% CIs: 1.55–1.82), while the cumulative OR for a 10 mm increase above the 75th percentile on WNND risk was 1.65 (95% CIs: 1.41–1.94). The attributable fraction (AF) of WNND cases for different thresholds of the two meteorological variables are listed in Table 1. Of all WNND cases recorded in the study period, 36.4% (95% empirical Confidence Intervals (eCIs): 31.3%–40.3%) were attributable to values above the 90th percentile of weekly mean temperature of the transmission season (25 °C), while 13.1% (95% eCI: 9.5%–16.4%) to values above the 90th percentile of weekly cumulative precipitation (40 mm).

**Table 1:** Attributable fractions (%) and 95% CIs of WNND cases for different thresholds of the meteorological factors.

Temperature (ºC)			Precipitation (mm)		
Threshold	AF (%)	95% eCI	Threshold	AF	95% eCI
75th (23.0)	Ref	–	75th (23.0)	Ref	–
90th (25.0)	36.4	31.3–40.3	90th (40)	13.1	9.5–16.4
95th (26.5)	22.0	18.3–25.2	95th (53)	7.5	5.4–9.5
99th (28.5)	6.2	4.7–7.3	99th (87)	0.6	0.4–0.8

AF: attributable fraction; eCIs: empirical confidence intervals.

**Fig. 4:**
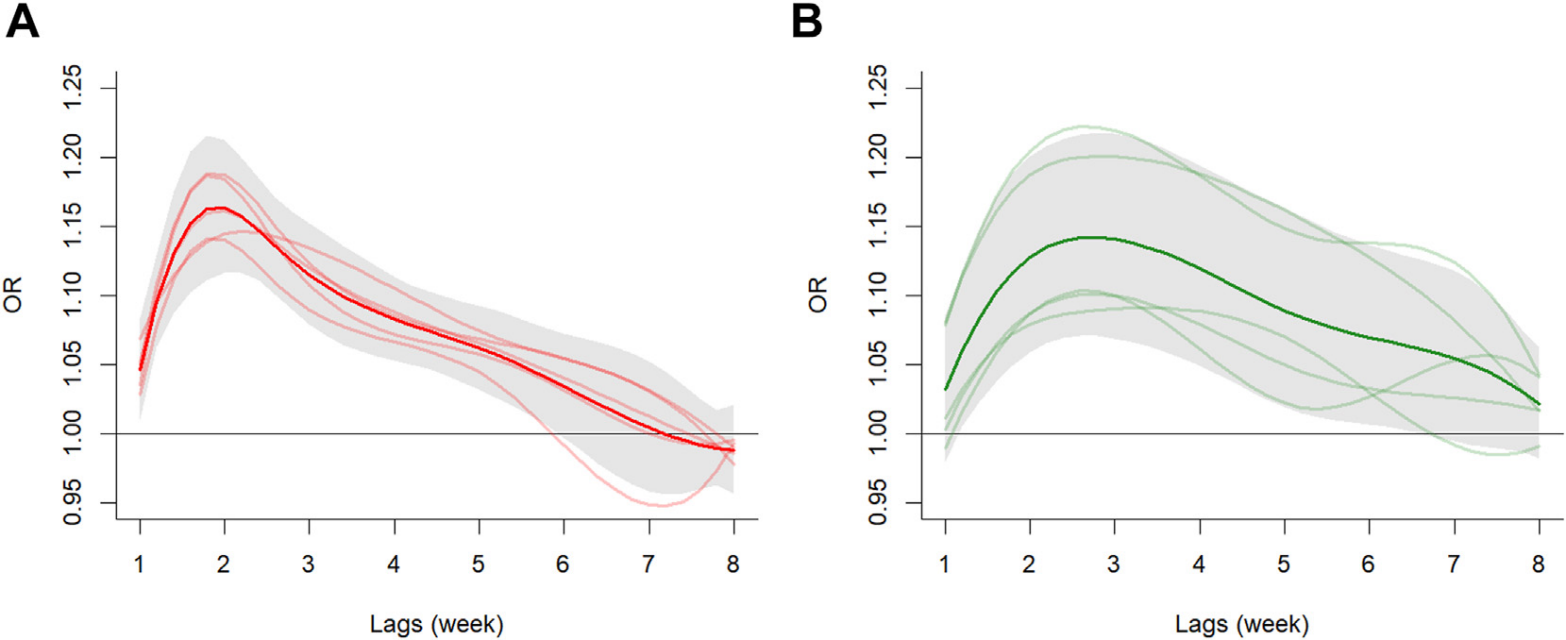
**Country-specific association between meteorological factors and WNND cases**. Panel A: Country-specific ORs (light red lines) for a 1 °C increase above the country-specific 75th percentile of weekly mean temperatures over 8 weeks of lag and pooled estimate (dark red line) and relative 95% CIs (grey areas) of all countries combined over 8 weeks of lag. Panel B: Country-specific ORs (light green lines) for a 10 mm increase above the country-specific 75th percentile of weekly cumulative precipitation over 8 weeks of lag and pooled estimates (dark green line) and relative 95% CIs (grey areas) of all countries combined over 8 weeks of lag.

In Fig. 4, country-specific (Italy, Greece, Romania, Serbia, Hungary) and meta-analytic estimates between lagged meteorological factors and WNND risk are shown. We did not observe any evidence of strong heterogeneity of the effect for temperature across the countries, with an I-squared statistic equal to 18%. Conversely the I-squared statistic for precipitation was equal to 31%, suggesting moderate heterogeneity of effects across the countries. Country specific BLUPs are reported in the Supplementary materials (Figures S3 and S4).

Results for the sensitivity analyses are reported in the Supplementary materials. Sensitivity analyses adjusted for seasonality decreased the overall effect for both meteorological parameters. However, the peak effect of temperature and precipitation was still observed at lag 2 and lag 3 weeks, respectively (Figure S5). Analyses conducted with the exclusion of outbreak years 2018 or 2022 provided consistent estimates with the main analyses (Figures S6 and S7).

## Discussion

Our analysis detected a positive delayed association between both meteorological factors and WNND risk. Specifically, high values of weekly mean temperatures were associated with an increased risk of WNND, where delayed effects ranged from 1 to 6 weeks with a peak at 2 weeks prior to symptoms onset. The influence of high ambient temperature on the number of WNND cases could be attributed to vector population dynamics (Culex species). Vector population abundance, for instance, is influenced by several biotic and abiotic factors.^7^ Among abiotic factors, temperature has been shown to be a strong determinant of *Culex pipiens* abundance both in laboratory experiments and in field data collected across different European areas.^18^ Our analysis detected a positive delayed association between both meteorological factors and WNND risk. Temperatures might also increase the risk of human infection by increasing the viral replication rate within the vector, amplifying the bird-mosquito transmission cycle and increasing the proportion of infected mosquiteos.^19^^,^^20^ In addition, previous studies showed that higher temperatures were followed by earlier and longer vector seasons.^21^^,^^22^ Concerning the incidence of human WNV cases in relation to temperature, several studies have shown that seasonal anomalies can increase the risk of human WNV cases among different years.^4^^,^^8^ Further, in studies that focused on the short-term effects of temperature, weekly temperatures were found to be positively associated with the delayed incidence of human WNV cases, including Romania,^9^ Greece,^23^ Italy,^24^ Israel,^25^ and in the US.^26^

In our study, we also observed that increased weekly cumulative precipitation was associated with an increased risk of WNND with a delayed effect spanning from 2 to 7 weeks, with a peak at lag 3 weeks. Many studies reported precipitation to be a key determinant of vector presence and abundance as it creates and expands aquatic habitats essential for mosquito reproductive cycle.^27^^,^^28^ However, it has been found that the amount of rainfall both in the same and previous months is associated with a decreased *Culex* abundance, suggesting that eggs and larvae might be washed out from their aquatic habitats by the water overflow both in urban and natural areas.^29^^,^^30^ In addition, some studies have suggested that drought periods can induce outbreaks favoring bird-to-bird viral transmission by facilitating the concentration of avian and mosquito species in the few existing pools.^31^ This uncertainty surrounding the role of precipitation on WNV dynamics is also reflected by research analysing human cases. For instance, some studies reported a positive association between increased cumulative precipitation and human WNV infection,^24^^,^^26^ while others reported no evidence or a negative association.^7^ It has been argued that the response to precipitation may vary over geographical areas, depending on the differences in the local environment and in the vector's ecology. Accordingly, our study observed a moderate degree of heterogeneity of the precipitation effects across the different European countries.

Our study has limitations. Firstly, data on the location of case occurrences were not available at a higher resolution than the NUTS3 level. Thus, we attributed to each case the temperature and precipitation recorded in the previous 8 weeks by aggregating ERA5-land reanalysis data on NUTS3 level. This linkage may have introduced some non-negligible degree of exposure misclassification. However, since case-crossover analysis uses the same subject as both case and control, misclassification is likely to be non-directional and lead to conservative estimates. Secondly, WNV diagnosis and surveillance might be differential both across the same season (i.e., WNV screening is more likely in symptomatic subjects after the first case of the season is diagnosed) and across different countries (i.e., endemic countries are more likely to diagnose WNV cases than non-endemic countries). Therefore, we decided to include only cases affected by WNND, which is a more severe clinical form of WNV, since serious neurological symptoms are more likely to be detected and less prone to underreporting. In addition, while our model accounts for the additive effects of temperature and precipitation, it does not explore potential interacting effects between these meteorological factors. Future studies may consider alternative modelling approaches to capture possible interactions between the two meteorological factors across multiple time lags. Lastly, it is important to acknowledge that estimates of the attributable fraction should be interpreted with caution, as they inherently assume a causal relationship between the exposure variables and the incidence of WNND cases. One of the strengths of this study is the application of a case-crossover design that, by using self-matching design, eliminates confounding associated with differences in constant characteristics, measured or unmeasured, typical of traditional case control studies. In addition, we conditioned the case-crossover sampling scheme by NUTS3 area, thus performing intra-area comparisons and therefore potential geographical confounders don't have to be measured and controlled for. Finally, we applied a time-stratified sampling scheme to account for the seasonality of both exposure and outcome. Furthermore, in sensitivity analyses we adjusted the model including a seasonal term (namely a spline function of day of the year) to further adjust estimates for the seasonal effect, obtaining consistent results, especially concerning the peak effect.

To our knowledge, this is the first study addressing the short-term effect of temperature and precipitation on all WNND cases diagnosed across the European continent (20 countries) over a relatively long period (9 years, 2014–2022). We adopted state-of-the-art methods to disentangle the complex relationships between meteorological factors and WNND incidence in Europe, where a non-negligible 36% and 13% of cases were attributable to weekly mean temperature and cumulative precipitation above the 90th percentile, respectively. These results are of interest when considering current climate change scenarios. A climate health attribution study reported that climate change directly increased local circulation of WNV in Europe and highlighted the responsibility of climate change in the establishment of WNV in the south-eastern part of the continent.^14^ In addition, our study paid specific attention to estimating the lag-effects of the meteorological variables, which can provide insights on the intra-seasonal delayed effect of meteorological factors on the count of WNND cases. For example, identifying the best time-lag that elapses between meteorological predictors and the outcome of interest can provide useful information for intra-seasonal disease forecasting.^32^ Our results indicate that these meteorological data are necessary to explain WNND risk in Europe. Since vector and host surveillance is labor, time and resource intensive, particularly on a continental scale, these findings have profound implications for preparedness and response. WNND early warning systems can be operationalized also in areas where biotic factors are not available. Developing such a timely system could have far-reaching public health implications, particularly in underfunded regions.

In conclusion, our results suggest that increased temperatures and precipitation have triggered the incidence of WNND cases in the following weeks and caused a not-negligible proportion of cases. These results strengthen the evidence that WNND is a climate-sensitive disease in Europe and that these meteorological signals can be used to operationalize early warning systems to reduce the disease burden from WNV infections, which are continually increasing across the continent.

## Contributors

Conceptualization: GM, RL; Data curation: GM; Formal analysis: GM; Funding acquisition: RL; Investigation: GM, CF, JS, RL; Methodology: GM, RL; Software: GM, CF, RL; Supervision: JS, RL; Validation: CF, RL; Visualization: GM, CF, JS, RL; Writing—original draft: GM, JS, RL; Writing– review & editing: GM, CF, JS, RL.

## Data sharing statement

Human cases data are available upon request at The European Surveillance System (https://www.ecdc.europa.eu/en/publications-data/european-surveillance-system-tessy). Meteorological data are available at the Copernicus Climate Change Service (https://climate.copernicus.eu/). An example dataset and a sample of the code to reproduce the analysis is available: https://earth.bsc.es/gitlab/ghr/wnv_casecrossover.

## Declaration of interests

Nothing to Declare.
