# Supplementary material for: Short-term effect of temperature and precipitation on the incidence of West Nile Neuroinvasive Disease in Europe: a multi-country case-crossover analysis
Source: Lancet Reg Health Eur. 2024 Dec 4;48:101149. doi: 10.1016/j.lanepe.2024.101149 (PMC11665362; doi:10.1016/j.lanepe.2024.101149)
Supplement: Supplementary Figures and Tables [file mmc1.docx]

**SUPPLEMENTARY MATERIALS**

**Table S1. Number of WNND cases by country over the study period, 2014-2022.**

| **Country** | **N of cases** | **Proportion** | **Cumulative Proportion** |
| --- | --- | --- | --- |
| Italy | 800 | 23.3 | 23.3 |
| Greece | 757 | 22.0 | 45.3 |
| Serbia | 723 | 21.0 | 66.3 |
| Romania | 615 | 17.9 | 84.2 |
| Hungary | 266 | 7.7 | 91.9 |
| Spain | 83 | 2.4 | 94.4 |
| Croatia | 59 | 1.7 | 96.1 |
| Turkey | 28 | 0.8 | 96.9 |
| Bulgaria | 19 | 0.5 | 97.4 |
| Cyprus | 19 | 0.5 | 97.9 |
| Germany | 19 | 0.5 | 98.4 |
| Austria | 12 | 0.3 | 98.7 |
| France | 11 | 0.3 | 99.0 |
| N. Macedonia | 9 | 0.3 | 99.3 |
| Netherlands | 6 | 0.2 | 99.5 |
| Czech Republic | <5 | <0.1 | 99.6 |
| Slovenia | <5 | <0.1 | 99.7 |
| Albania | <5 | <0.1 | 99.8 |
| Portugal | <5 | <0.1 | 99.9 |
| Slovakia | <5 | <0.1 | 100.00 |
| **Total** | **3,437** | **100.00** | **100.00** |

**Table S2. Overall and country specific distribution of weekly average temperature and weekly cumulative precipitation between May and October including all NUTS3 areas that reported WNND cases in the study period, 2014-2022, and stratified by country.**

|  | **Temperature (ºC)** | | | **Precipitation (mm)** | | |
| --- | --- | --- | --- | --- | --- | --- |
| **Country** | **P25** | **Median** | **P75** | **P25** | **Median** | **P75** |
| Albania | 15.2 | 19.5 | 22.1 | 4.4 | 11.3 | 34.1 |
| Austria | 14.2 | 18.7 | 21.6 | 6.1 | 13.2 | 23.0 |
| Bulgaria | 15.1 | 19.3 | 22.1 | 3.8 | 9.7 | 20.7 |
| Croatia | 15.2 | 19.6 | 22.6 | 6.6 | 17.2 | 21.9 |
| Cyprus | 23.4 | 25.9 | 28.7 | 0.0 | 0.0 | 1.8 |
| Czech Republic | 13.2 | 17.6 | 20.5 | 6.6 | 13.4 | 21.9 |
| France | 15.9 | 19.7 | 22.4 | 5.3 | 10.1 | 20.3 |
| Germany | 13.7 | 17.2 | 19.7 | 6.3 | 13.0 | 20.8 |
| Greece | 18.2 | 22.2 | 25.1 | 2.2 | 6.4 | 13.0 |
| Hungary | 15.1 | 19.7 | 22.6 | 5.2 | 13.5 | 22.0 |
| Italy | 16.1 | 19.9 | 22.7 | 10.0 | 19.6 | 32.9 |
| N. Macedonia | 14.8 | 19.0 | 22.0 | 2.3 | 9.1 | 17.4 |
| Netherlands | 13.8 | 16.5 | 18.4 | 5.0 | 14.1 | 24.6 |
| Portugal | 20.2 | 22.2 | 24.6 | 0.0 | 0.0 | 2.8 |
| Romania | 14.0 | 18.8 | 21.6 | 5.1 | 13.8 | 23.3 |
| Serbia | 15.3 | 19.6 | 22.2 | 4.9 | 11.8 | 23.1 |
| Slovakia | 14.2 | 18.8 | 21.8 | 4.0 | 11 | 21 |
| Slovenia | 13.6 | 17.4 | 20.3 | 10.1 | 23.3 | 39.9 |
| Spain | 18.2 | 22.2 | 25.1 | 0.8 | 6.4 | 13.0 |
| Turkey | 17.5 | 21.0 | 24.2 | 2.3 | 6.1 | 11.9 |
| **Overall** | **15.6** | **19.5** | **22.8** | **2.3** | **9.9** | **23.0** |

T = Weekly average Temperature recorded between June and October; Prep = Weekly cumulative precipitation between June and October. p25= 25° percentile, p50 = 50° percentile, p75= 75° percentile.

**Table S3. List of univariable and multivariable DLNM models fitted in the exploratory analyses with corresponding AIC values.**

| **Weekly Mean Temperature** | | **Weekly Cumulative Precipitation** | |  |
| --- | --- | --- | --- | --- |
| **Exposure-Response Function** | **Lag Function** | **Exposure-Response Function** | **Lag Function** | **AIC** |
| f(x): Linear | f(*l*): Basis Cubic Spline  knots(*l*): 2.66, 5.32 |  |  | 9832 |
| f(x): Linear | f(*l*): Natural Cubic Spline  knots(*l*): 2.66, 5.32 |  |  | 9837 |
| f(x): Natural Cubic Spline  knots(*x*): 16ºC, 23ºC | f(*l*): Basis Cubic Spline  knots(*l*): 2.66, 5.32 |  |  | 9813 |
| f(x): Natural Cubic Spline  knots(*x*): 16ºC, 23ºC | f(*l*): Natural Cubic Spline  knots(*l*): 2.66, 5.32 |  |  | 9812 |
| f(x): Natural Cubic Spline  knots(*x*): 17ºC, 22ºC | f(*l*): Basis Cubic Spline  knots(*l*): 2.66, 5.32 |  |  | 9811 |
| f(x): Natural Cubic Spline  knots(*x*): 17ºC, 22ºC | f(*l*): Natural Cubic Spline  knots(*l*): 2.66, 5.32 |  |  | 9810 |
|  |  | f(x): Linear | f(*l*): Basis Cubic Spline”  knots(*l*): 2.66, 5.32 | 10186 |
|  |  | f(x): Linear | f(*l*): Natural Cubic Spline”  knots(*l*): 2.66, 5.32 | 10198 |
|  |  | f(x): Natural Cubic Spline  knots(*x*): 2 mm, 23 mm | f(*l*): Basis Cubic Spline”  knots(*l*): 2.66, 5.32 | 10192 |
|  |  | f(x): Natural Cubic Spline  knots(*x*): 2 mm, 23 mm | f(*l*): Natural Cubic Spline”  knots(*l*): 2.66, 5.32 | 10202 |
|  |  | f(x): Natural Cubic Spline  knots(*x*): 4 mm, 17 mm | f(*l*): Basis Cubic Spline”  knots(*l*): 2.66, 5.32 | 10190 |
|  |  | f(x): Natural Cubic Spline  knots(*x*)): 4 mm, 17 mm | f(*l*): Natural Cubic Spline”  knots(*l*): 2.66, 5.32 | 10201 |
| f(x): Natural Cubic Spline  knots(*x*): 16ºC, 23ºC | f(*l*): Natural Cubic Spline  knots(*l*): 2.66, 5.32 | f(x): Linear | f(*l*): Basis Cubic Spline”  knots(*l*): 2.66, 5.32 | 9762 |
| f(x): Natural Cubic Spline  knots(*x*): 16ºC, 23ºC | f(*l*): Natural Cubic Spline  knots(*l*): 2.66, 5.32 | f(x): Natural Cubic Spline  knots(*x*): 2 mm, 23 mm | f(*l*): Basis Cubic Spline”  knots(*l*): 2.66, 5.32 | 9755 |
| f(x): Natural Cubic Spline  knots(*x*): 16ºC, 23ºC | f(*l*): Natural Cubic Spline  knots(*l*): 2.66, 5.32 | f(x): Natural Cubic Spline  knots(*x*)): 4 mm, 17 mm | f(*l*): Natural Cubic Spline”  knots(*l*): 2.66, 5.32 | 9761 |
| **f(x): Natural Cubic Spline**  **knots(*x*): 17ºC, 22ºC** | **f(*l*): Basis Cubic Spline**  **knots(*l*): 2.66, 5.32** | **f(x): Linear** | **f(*l*): Basis Cubic Spline”**  **knots(*l*): 2.66, 5.32** | **9754** |
| f(x): Natural Cubic Spline  knots(*x*): 17ºC, 22ºC | f(*l*): Basis Cubic Spline  knots(*l*): 2.66, 5.32 | f(x): Natural Cubic Spline  knots(*x*): 2 mm, 23 mm | f(*l*): Natural Cubic Spline”  knots(*l*): 2.66, 5.32 | 9761 |
| f(x): Natural Cubic Spline  knots(*x*): 17ºC, 22ºC | f(*l*): Basis Cubic Spline  knots(*l*): 2.66, 5.32 | f(x): Natural Cubic Spline  knots(*x*)): 4 mm, 17 mm | f(*l*): Natural Cubic Spline”  knots(*l*): 2.66, 5.32 | 9760 |
| f(x): Natural Cubic Spline  knots(*x*): 17ºC, 22ºC | f(*l*): Natural Cubic Spline  knots(*l*): 2.66, 5.32 | f(x): Linear | f(*l*): Basis Cubic Spline”  knots(*l*): 2.66, 5.32 | 9754 |
| f(x): Natural Cubic Spline  knots(*x*): 17ºC, 22ºC | f(*l*): Natural Cubic Spline  knots(*l*): 2.66, 5.32 | f(x): Natural Cubic Spline  knots(*x*): 2 mm, 23 mm | f(*l*): Natural Cubic Spline”  knots(*l*): 2.66, 5.32 | 9761 |
| f(x): Natural Cubic Spline  knots(*x*): 17ºC, 22ºC | f(*l*): Natural Cubic Spline  knots(*l*): 2.66, 5.32 | f(x): Natural Cubic Spline  knots(*x*)): 4 mm, 17 mm | f(*l*): Natural Cubic Spline”  knots(*l*): 2.66, 5.32 | 9760 |

f(*x*): function describing the exposure-response relationship, knots*(x):* position of knots for spline function across the range of exposure *x*. f(*l*): function describing the lag-response relationship, knots*(l):* position of knots for spline function over the lags (1-8 weeks). AIC: Akaike Information Criterion.

**Table S4. Lag-specific ORs and 95% confidence intervals for weekly average temperature and weekly cumulative precipitation and WNND incident cases in the study area, 2014-2022.**

|  | **Temperature** |  | **Precipitation** | |
| --- | --- | --- | --- | --- |
| **Lag (weeks)** | **OR^a^** | **95% CIs** | **OR^b^** | **95% CIs** |
| 1 | 1.08 | 1.05 - 1.10 | 1.02 | 0.99 - 1.05 |
| 2 | 1.15 | 1.12 - 1.19 | 1.10 | 1.08 - 1.14 |
| 3 | 1.12 | 1.09 - 1.14 | 1.12 | 1.09 - 1.16 |
| 4 | 1.09 | 1.07 - 1.11 | 1.10 | 1.07 - 1.13 |
| 5 | 1.07 | 1.05 - 1.09 | 1.07 | 1.04 - 1.10 |
| 6 | 1.04 | 1.02 - 1.05 | 1.05 | 1.02 - 1.08 |
| 7 | 1.00 | 0.98 - 1.03 | 1.04 | 1.00 - 1.07 |
| 8 | 1.00 | 0.97 - 1.02 | 1.01 | 0.98 -1.04 |
| **Cumulative Effect** | 1.68 | 1.55 – 1.82 | 1.65 | 1.41 - 1.94 |

^a^OR for a 1°C increase of weekly average temperature above the 75^th^ percentile (23°C).

^b^OR for 10 mm increase in weekly cumulative precipitation.

**
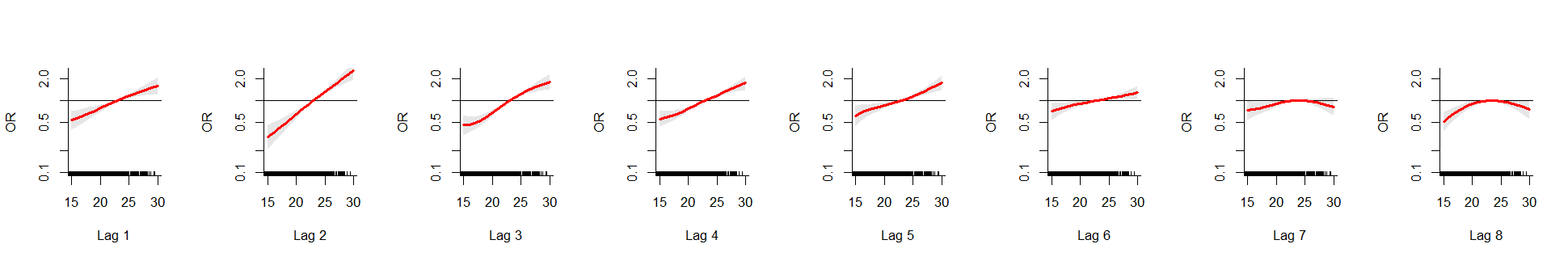
**

**Fig S1. Lag-specific exposure-response function for Weekly Average Temperatures (°C) and WNND incident cases (N=3,437) in the study area, 2014-2022.**

OR (red lines) and 95% CIs (grey areas) for lag-specific exposure-response function.


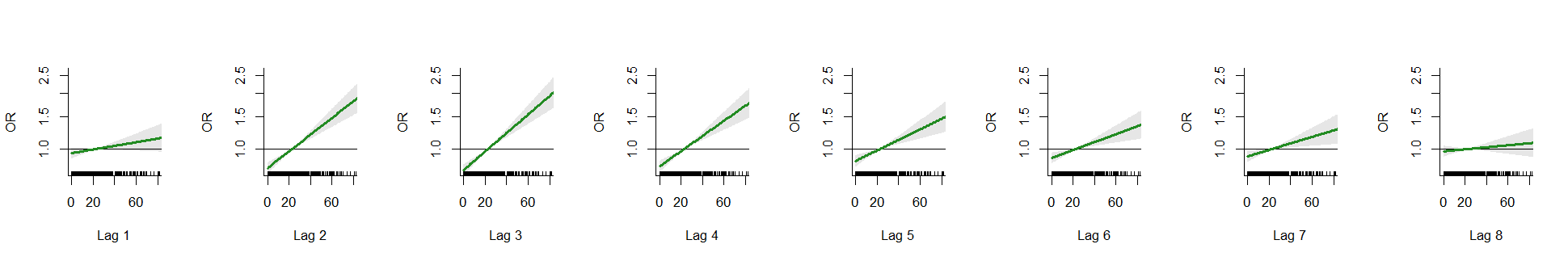


**Fig S2. Lag-specific exposure-response function for Weekly Cumulative Precipitation (mm) and WNND incident cases (N=3,437) in the study area, 2014-2022.**

OR (green lines) and 95% CIs (grey areas) for lag-specific exposure-response function.


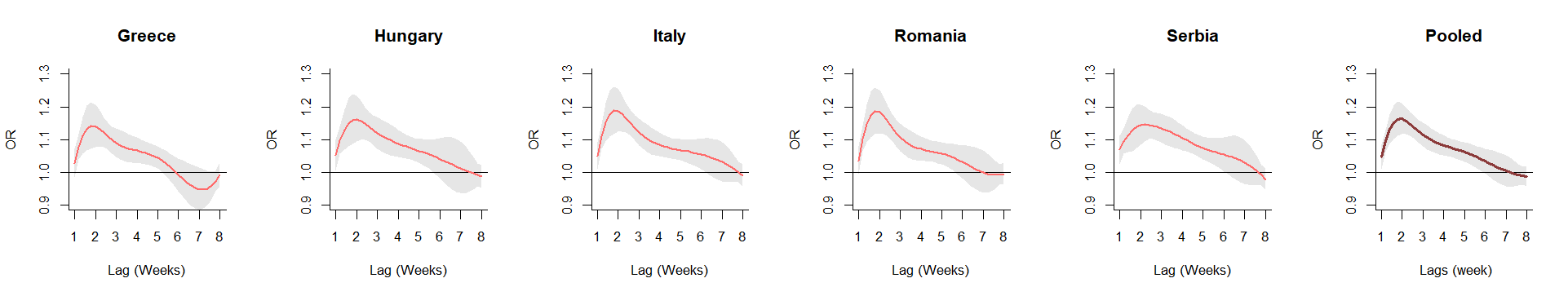


**Fig.S3 Country-specific association between Weekly Average Temperature (°C) and WNND incident cases, 2014-2022.**

Lag-specific OR (red lines) and 95% CIs (grey areas) for a 1°C increase above the country-specific 75^th^ percentile of weekly mean temperatures (23ºC) over 8 weeks of lag. Pooled: Pooled estimates of all countries combined over 8 weeks of lag.


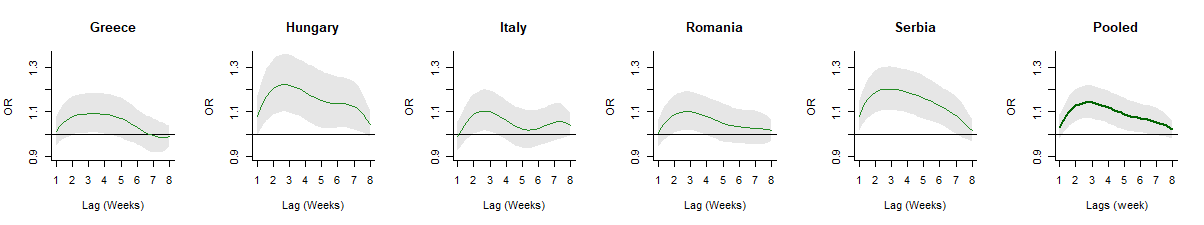


**Fig.S4 Country-specific association between Weekly Cumulative Precipitation (mm) and WNND incident cases, 2014-2022.**

Lag-specific OR (green lines) and 95% CIs (grey areas) for a 1°C increase above the country-specific 75^th^ percentile of weekly mean temperatures (23ºC) over 8 weeks of lag. Pooled: Pooled estimates of all countries combined over 8 weeks of lag.


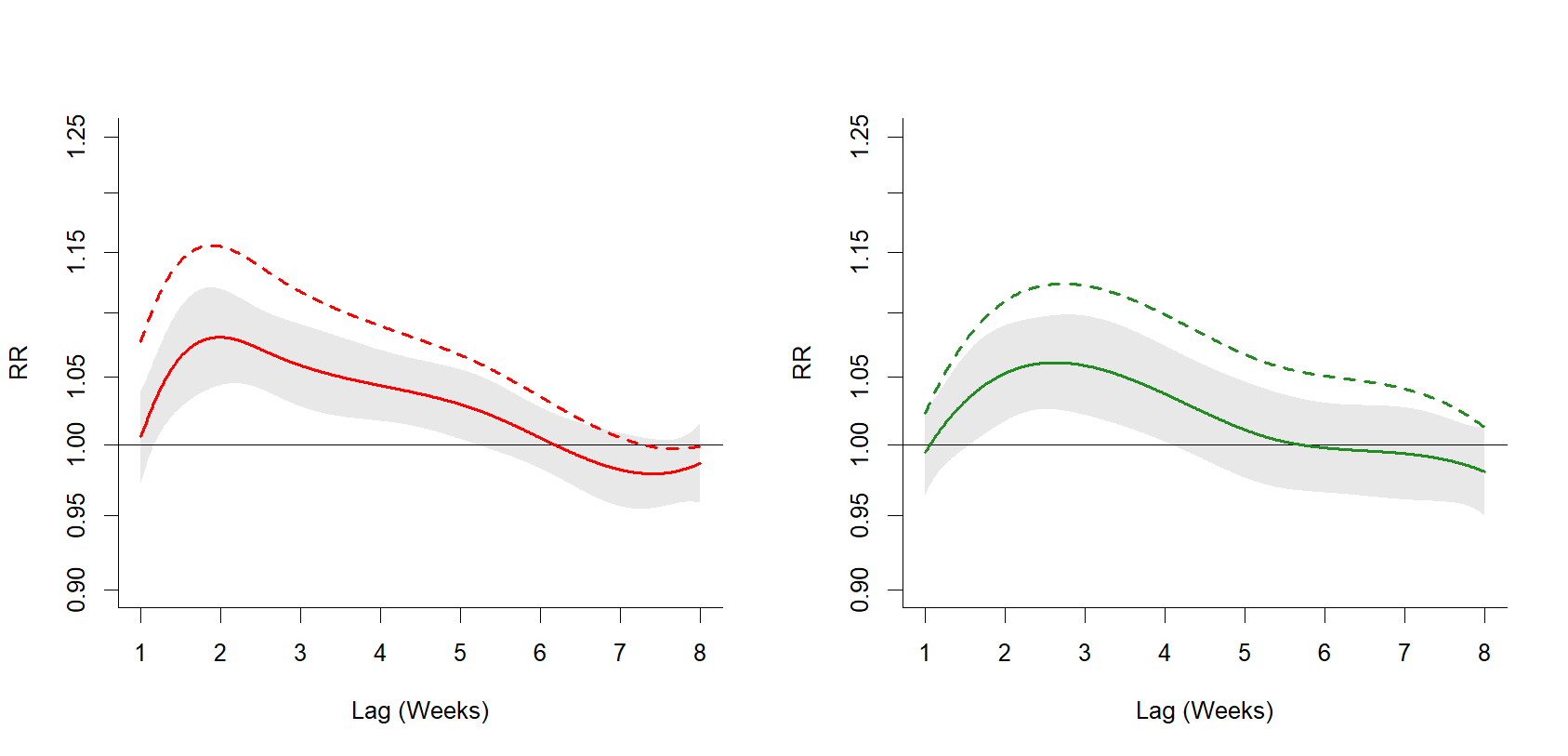


**Fig S5. Sensitivity analyses additionally adjusted for seasonal pattern (cubic spline of day of the year with 7 equally spaced knots).**

Left Panel: Lag-specific OR (continuous line) and 95% confidence intervals (grey area) for a 1°C increase above the 75^th^ percentile (23°C) over 8 weeks of lag among WNND cases adjusted for seasonal pattern; Lag-specific OR (dashed line) for a 1°C increase above the 75^th^ percentile (23°C) over 8 weeks of lag among all WNND cases (Main Analyses). Right Panel: Lag-specific OR (continuous line) and 95% confidence intervals (grey area) for a 10 mm increase above the 75^th^ percentile (23 mm) over 8 weeks of lag among all WNND cases adjusted for seasonal pattern; Lag-specific (dashed line) for a 10 mm increase above the 75^th^ percentile (23 mm) over 8 weeks of lag among all WNND cases adjusted (Main analyses).

**
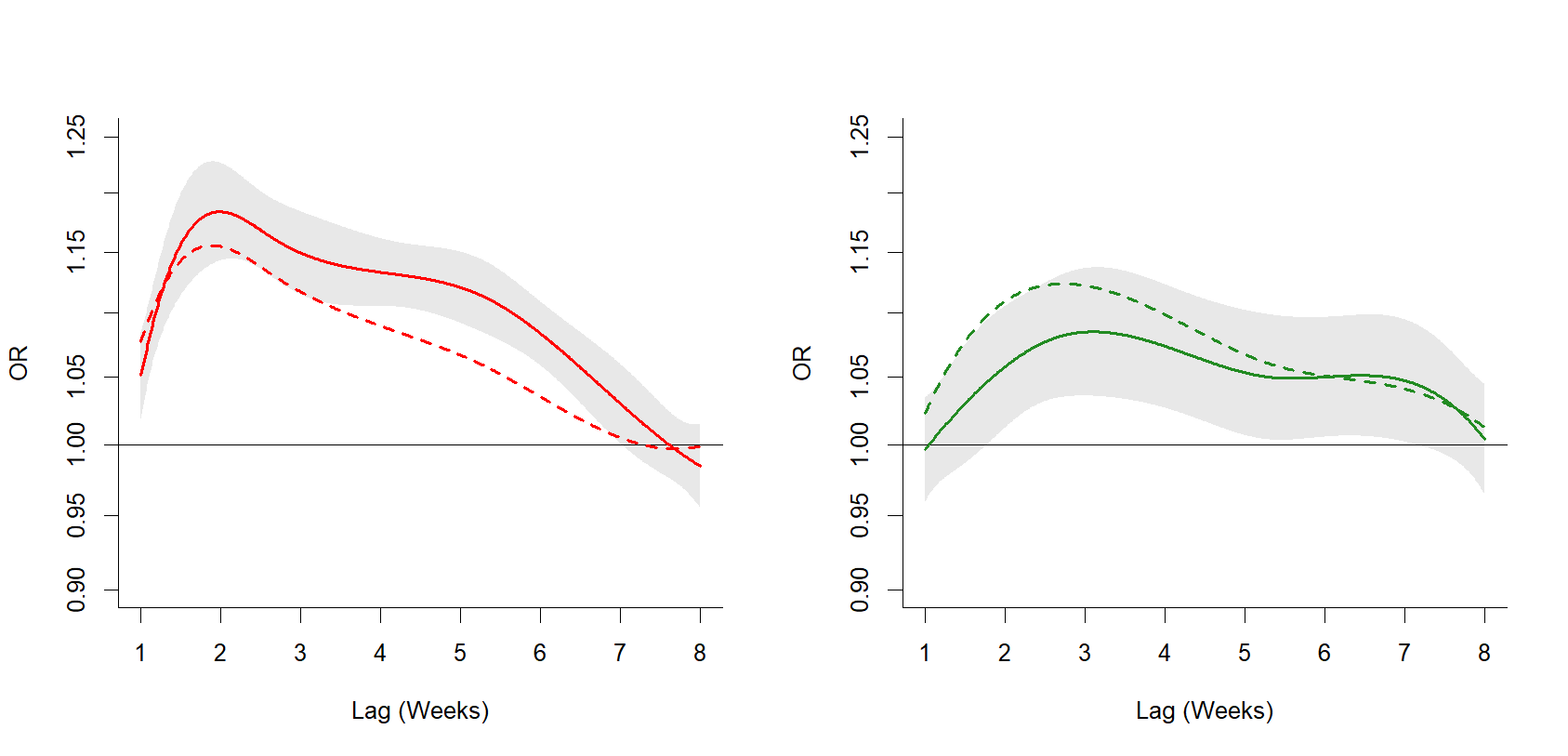
**

**Fig S6. Sensitivity analyses excluding 2018 WNND cases.**

Left Panel (A): Lag-specific OR (continuous line) and 95% CIs (grey area) for a 1°C increase above the 75^th^ percentile (23°C) over 8 weeks of lag among WNND cases excluding 2018; Lag-specific OR (dashed line) for a 1°C increase above the 75^th^ percentile (23°C) over 8 weeks of lag among all WNND cases (Main Analyses). Right Panel (B): Lag-specific OR (continuous line) and 95% CIs (grey area) for a 10 mm increase above the 75^th^ percentile (23 mm) over 8 weeks of lag among all WNND cases excluding 2018; Lag-specific OR (dashed line) for a 10 mm increase above the 75^th^ percentile (23 mm) over 8 weeks of lag among all WNND cases (Main analyses).

**
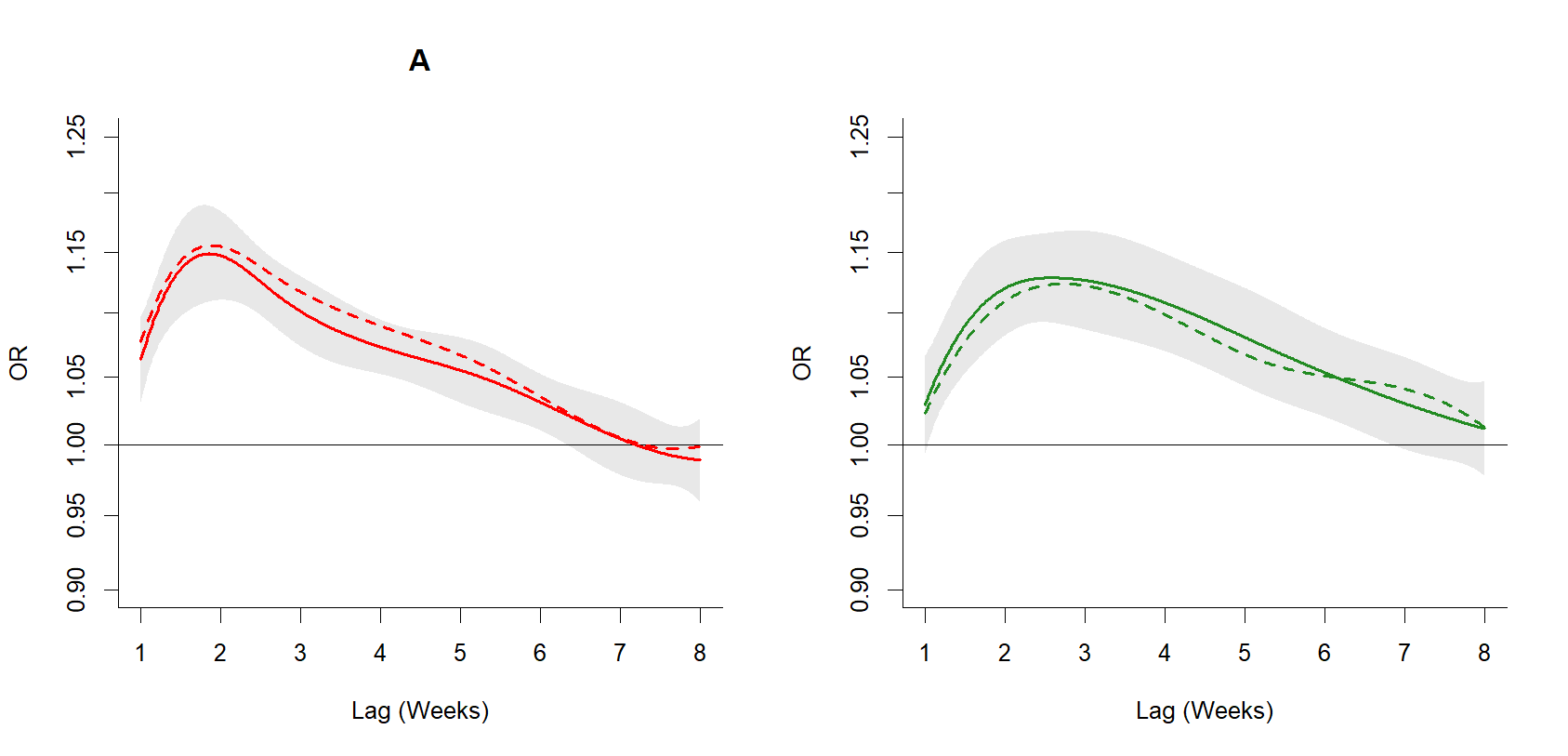
**

**Fig S7. Sensitivity analyses excluding 2022 WNND cases.**

Left Panel: Lag-specific OR (continuous line) and 95% CIs (grey area) for a 1°C increase above the 75^th^ percentile (25°C) over 8 weeks of lag among WNND cases excluding 2022; Lag-specific OR (dashed line) for a 1°C increase above the 75^th^ percentile (23°C) over 8 weeks of lag among all WNND cases (Main Analyses). Right Panel: Lag-specific OR (continuous line) and 95% CIs (grey area) for a 10 mm increase above the 75^th^ percentile (23 mm) over 8 weeks of lag among all WNND cases excluding 2018; Lag-specific OR (dashed line) for a 10 mm increase above the 75^th^ percentile (23 mm) over 8 weeks of lag among all WNND cases (Main analyses).
